# CTD anatomy: Analyzing chemical-induced phenotypes and exposures from an anatomical perspective, with implications for environmental health studies

**DOI:** 10.1016/j.crtox.2021.03.001

**Published:** 2021-03-05

**Authors:** Allan Peter Davis, Thomas C. Wiegers, Jolene Wiegers, Cynthia J. Grondin, Robin J. Johnson, Daniela Sciaky, Carolyn J. Mattingly

**Affiliations:** a Department of Biological Sciences, North Carolina State University, Raleigh, NC 27695, United States; b Center for Human Health and the Environment, North Carolina State University, Raleigh, NC 27695, United States

**Keywords:** Anatomy, Chemical, Phenotype, Disease, Environmental health, Database, CGPD, chemical-gene-phenotype-disease, CIN, chemical inference network, CL, Cell Ontology, CTD, Comparative Toxicogenomics Database, GIN, gene inference network, GO, Gene Ontology, ID, identifier, MeSH, Medical Subject Headings, PCB, polychlorinated biphenyl, PBDE, polybromodiphenyl ether

## Abstract

The Comparative Toxicogenomics Database (CTD) is a freely available public resource that curates and interrelates chemical, gene/protein, phenotype, disease, organism, and exposure data. CTD can be used to address toxicological mechanisms for environmental chemicals and facilitate the generation of testable hypotheses about how exposures affect human health. At CTD, manually curated interactions for chemical-induced phenotypes are enhanced with anatomy terms (tissues, fluids, and cell types) to describe the physiological system of the reported event. These same anatomy terms are used to annotate the human media (e.g., urine, hair, nail, blood, etc.) in which an environmental chemical was assayed for exposure. Currently, CTD uses more than 880 unique anatomy terms to contextualize over 255,000 chemical-phenotype interactions and 167,000 exposure statements. These annotations allow chemical-phenotype interactions and exposure data to be explored from a novel, anatomical perspective. Here, we describe CTD’s anatomy curation process (including the construction of a controlled, interoperable vocabulary) and new anatomy webpages (that coalesce and organize the curated chemical-phenotype and exposure data sets). We also provide examples that demonstrate how this feature can be used to identify system- and cell-specific chemical-induced toxicities, help inform exposure data, prioritize phenotypes for environmental diseases, survey tissue and pregnancy exposomes, and facilitate data connections with external resources. Anatomy annotations advance understanding of environmental health by providing new ways to explore and survey chemical-induced events and exposure studies in the CTD framework.

## Highlights

• Anatomy terms are used to contextualize chemical-induced outcomes.
• CTD Anatomy analysis can aid understanding of environmental disease mechanisms.
• Examples show how to survey chemical toxicities and support exposome research.

## Graphical abstract

**Figure:**
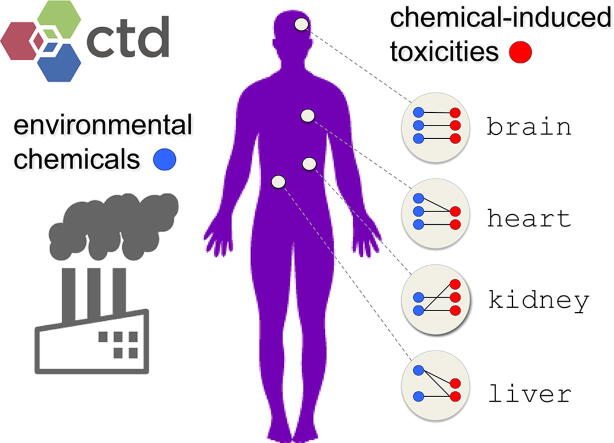

## 1 Introduction

The Comparative Toxicogenomics Database (CTD; http://ctdbase.org/) provides information that advances the understanding of how environmental exposures affect human heath (Davis et al., 2021). Since 2004, CTD has spearheaded the manual curation of environmental chemical data from the scientific literature by coding and contextualizing chemical-gene, chemical-disease, gene-disease, chemical-phenotype, and chemical-exposure interactions in a structured format using controlled vocabularies and ontologies (Davis et al., 2015). This helps standardize and harmonize information reported by different laboratories published in a variety of journals over the decades, makes the information computable, and facilitates data exchange with other resources. The use of controlled vocabularies enables CTD data to be computationally integrated internally with other curated data sets and with select external public data sets to generate inference networks connecting heterogeneous data types, which, in turn, can be used to construct testable hypotheses to elucidate environmental diseases (Davis et al., 2008).

We introduce CTD Anatomy as a vocabulary used to contextualize curated chemical-phenotype interactions and exposure data with tissue and cell descriptions to provide the anatomical context for the reported chemical-induced toxicities (Davis et al., 2013, Davis et al., 2018). Encoding anatomy details using a controlled vocabulary makes the information computable and adds yet another data type to act as a nexus for connecting with the diverse research community (Dahdul et al., 2015). Here, we describe the construction, interoperability, implementation, visualization, and incorporation of the CTD Anatomy module into CTD, including online tools. As well, we provide several examples demonstrating how CTD Anatomy content can be leveraged, especially with its integration to CTD Exposure, a curated module relating real-life environmental exposure study data and biomarker measurements (Grondin et al., 2016, Grondin et al., 2018), to help generate testable hypotheses and inform environmental health studies.

Gene-environment interactions are suggested to have a more substantial influence, compared to genes alone, on chronic human diseases (Rappaport, 2016), and chemical exposure is a key component of the environment, diet, and metabolism (Barabasi et al., 2020, Smith and Perfetti, 2020). Recently, numerous laboratories have successfully integrated CTD chemical content with genome-wide association studies to identify environmental chemicals associated with colorectal cancer (Tan et al., 2020), psychiatric disorders (Cheng et al., 2020), breast cancer (Gong et al., 2020), immune dysfunction (Wang et al., 2020), insomnia (Kafle et al., 2020), epilepsy (Brennan et al., 2020), and altered metabolic traits (Lu et al., 2020). Another critical component to understanding the development of human disease is the recognition of the diverse molecular and genetic milieu of different tissues, including the expression and interaction of tissue-specific gene networks, and how their regulated expression and restriction can relate to tissue-centric diseases (Kitsak et al., 2016, Gokhman et al., 2017). Thus, understanding how environmental chemicals interact in physiology-specific mechanisms at different anatomical sites should further inform the etiology of environmental diseases (Taboureau et al., 2020). We demonstrate how CTD Anatomy can be leveraged to help resolve knowledge gaps between environmental exposure and human health outcomes.

## 2 Materials and methods

### 2.1 Data version

Analysis was performed using CTD public data released November 2020 (revision 16353). CTD is updated with new content on a monthly basis (http://ctdbase.org/about/dataStatus.go); consequently, results derived in this text may vary over time.

### 2.2 CTD phenotypes vs. diseases

CTD curation paradigms, practices, and database load/publishing architecture have been previously described in detail (Davis et al., 2011). At CTD, “phenotypes” are defined as chemical-induced aberrations (e.g., toxicities) curated from the toxicology literature and are operationally distinguished from “diseases” (Davis et al., 2013), wherein a phenotype refers to a non-disease biological event: e.g., “decreased neuron differentiation” is a phenotype, while “amyotrophic lateral sclerosis” is a disease; “abnormal cardiac muscle cell proliferation” is a phenotype, while “myocardial ischemia” is a disease; “increased apoptosis” is a phenotype, while “microcephaly” is a disease, etc. Two separate vocabularies are used to curate this distinction: for diseases, CTD biocurators use terms from MEDIC (Davis et al., 2012), a MErged DIsease voCabulary that combines the terms, accessions, and gene-disease content from the Online Mendelian Inheritance in Man resource (Amberger et al., 2019) with the terms, accessions, and navigable hierarchy of Medical Subject Headings (MeSH) “Disease” branch (https://meshb.nlm.nih.gov/treeView); however, if the reported outcome does not exist as a term in MEDIC, then, by definition, it is considered a phenotype and curated using terms from the Gene Ontology (GO) (Ashburner et al., 2000) to describe the biological event and is constructed as a chemical-induced toxicity (Davis et al., 2018). Data integration is a key feature at CTD, allowing the discovery of novel data connections. Phenotypes can be inferred to diseases based upon shared chemicals and/or genes (Davis et al., 2013, Davis et al., 2020). Thus, if chemical C1 is reported to interact with phenotype P1 and independently with disease D1, then phenotype P1 can be inferred to disease D1 based upon a shared Chemical Inference Network (CIN) that includes C1. Similarly, if gene G1 is independently associated with both phenotype/GO term P1 and disease D1, then phenotype P1 can be inferred to disease D1 based upon a shared Gene Inference Network (GIN).

### 2.3 CTD Anatomy

To build a comprehensive, interoperable anatomical vocabulary, we started with descriptor and supplementary concept terms from MeSH “Anatomy [A]” (Coletti and Bleich, 2001). MeSH, as a controlled thesaurus, offers many practical advantages: it is freely available, reliable, and easy to programmatically access, being professionally maintained by the U.S. National Library of Medicine; it provides term definitions, synonyms, and stable accession identifiers allowing for data interoperability and FAIRness (Wilkinson et al., 2016); it is robust, including terms for physiological systems, tissues, fluids, cell types, and sub-cellular components; it is organized as a displayable, navigable hierarchy, facilitating easy data exploration and meta-analysis; and, importantly, it is used to index scientific articles in PubMed (Sayers et al., 2020), simplifying the querying/retrieval of scientific publications. We started with MeSH “Anatomy” tree branch (https://meshb.nlm.nih.gov/treeView) as the core; next, we computationally excluded several organism-specific sub-branches that relate to species not curate in CTD: “Plant Structures”, “Fungal Structures”, “Bacterial Structures”, and “Viral Structures”, yet retained the “Cellular Structures” sub-branch that includes component terms typically not found in other anatomical vocabularies (e.g., axons, nucleus, mitochondria, lysosome, adherens junctions, etc.). Other terms (1103) mapping to both “Anatomy” and “Disease” MeSH trees (e.g., Adactylia, Unilateral [MeSH:C562417]) were removed from CTD Anatomy (but retained in CTD MEDIC). CTD collects, stores, and displays the MeSH Heading (“Name”), Entry Term(s) (“Synonyms”), Scope Note (“Definition”), Unique ID (“MeSH ID”), Tree Numbers, and Parent Tree Numbers. To enhance its practicality and promote interoperability with other resources, we mapped these MeSH terms to two other anatomy vocabularies: Uberon (Mungall et al., 2012), which focuses exclusively on organs and tissues, and the Cell Ontology (CL), which is limited to in vivo cell-types (Diehl et al., 2016). MeSH terms were compared against Uberon (http://uberon.org) and CL (https://github.com/obophenotype/cell-ontology), independently, to look for potential term matches. A designed algorithm evaluated matches between vocabularies (MeSH vs. Uberon or CL) by four criteria: direct term-to-term match, MeSH term to Uberon/CL synonym match, MeSH synonym to Uberon/CL term match, and MeSH term accession to Uberon/CL cross-reference accession match. Matching results can differ based upon the order-of-use of the match algorithms; three different match order options were performed, and the outputs were manually reviewed, validated, and corrected when necessary to optimize mappings. Matching Uberon/CL terms (and their internal synonyms) were merged and made “External Synonyms” to the matching MeSH term; synonyms were only created in cases where they augmented the existing MeSH synonyms.

### 2.4 Data collection and analysis

CTD query (http://ctdbase.org/search/) and analyses tools (http://ctdbase.org/tools/) were used to retrieve and analyze all data sets. Chemical, phenotype, and exposure curation were collected from the relevant data-tabs on anatomy webpages-of-interest and transferred to spreadsheets using the “Download” feature at the bottom of CTD Anatomy webpages; data were sorted and filtered to find the number of unique terms. All CTD curated content is freely available as structured data files for downloading and programmatic analysis by users (http://ctdbase.org/downloads/)

For example 1: “Chemical-Phenotype Interactions” data-tabs on CTD Anatomy pages (accession ID) were downloaded for Liver (MeSH:D008099), Kidney (MeSH:D007668), Brain (MeSH:D001921), and Heart (MeSH:D006321); data associated with descendant terms were subsumed and used in the analysis. Unique chemical, phenotype, and chemical-phenotype dyads were compared using Venny 2.1 (https://bioinfogp.cnb.csic.es/tools/venny/index.html). Specific immune system cell terms or accession IDs were input into CTD’s Batch Query to retrieve associated chemical-phenotype data using the filter to return data for exact input term and any descendant. Data were transferred to spreadsheets and then sorted and filtered to find the number of unique chemicals and phenotypes for each cell type. For visualization, an edited, schematic diagram of the “Immune System” was drawn based upon its hierarchy in CTD Anatomy (http://ctdbase.org/detail.go?type=anatomy&acc=D007107), and the unique number of chemicals and phenotypes were overlaid for each surveyed immune cell. The top reported chemicals and phenotypes were broken out and graphed for each distinct immune cell type.

For example 2: “Chemical-Phenotype Interactions” and “Exposure Studies” data-tabs on CTD Anatomy page Tibia (MeSH:D013977) were used to combine bone-related phenotypes and bone-related exposure outcomes. The Inference Network provides gene sets connecting lead to the phenotypes of osteoclast and bone development. Next, “Phenotypes” data-tabs on CTD Disease pages were downloaded for Stomach Neoplasms (MeSH:D013274) and Autistic Disorder (MeSH:D001321), including phenotype data associated with their descendant disease terms, and then, independently, were sorted to collect only the phenotypes inferred by both a CIN and GIN. “Chemical-Phenotype Interactions” data-tabs on CTD Anatomy pages were downloaded for Stomach (MeSH:D013270), Neurons (MeSH:D009474), and Brain (MeSH:D001921), and lists of unique phenotypes for each anatomical term were resolved.

For example 3: “Exposure Studies” and “Exposure Details” data-tabs were downloaded, sorted, and analyzed to identify unique numbers of studies, measurements, chemicals, and genes for the following CTD Anatomy pages: Urine (MeSH:D014556), Serum (MeSH:D044967), Plasma (MeSH:D010949), Blood Cells (MeSH:D001773), Hair (MeSH:D006197), Saliva (MeSH:D012463), Adipose Tissue (MeSH:D000273), Semen (MeSH:D012661), Tears (MeSH:D013666), Sweat (MeSH:D013542), Fetal Blood (MeSH:D005312), Milk Human (MeSH:D008895), and Placenta (MeSH:D010920).

## 3 Results and discussion

### 3.1 CTD Anatomy

**Fig. 1:**
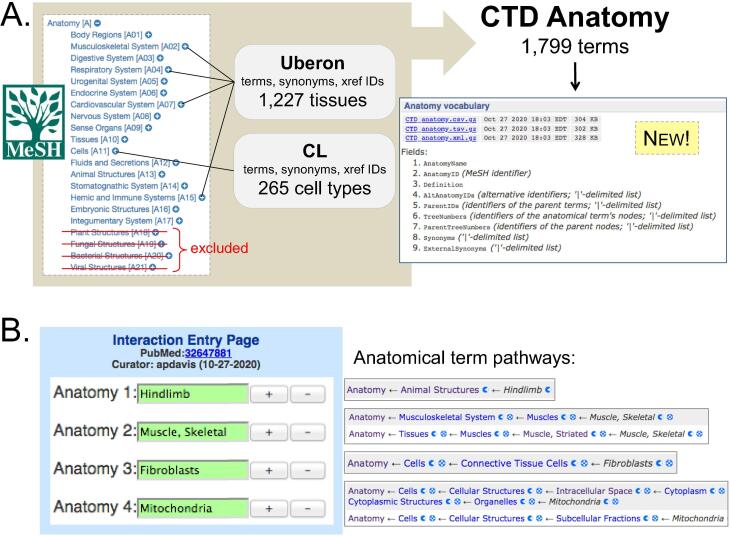
Building and implementing CTD Anatomy. (A) MeSH “Anatomy” files were downloaded and computationally edited to exclude organism-specific sub-branches for species not curated in CTD (plants, fungi, bacteria, and viruses). Next, the terms, synonyms and cross-reference accession identifiers (x-ref IDs) for 1227 organs/tissues from Uberon and 265 cell types from Cell Ontology (CL) were programmatically mapped (and then manually reviewed) into the vocabulary, building an interoperable resource that currently includes 1799 primary terms. CTD Anatomy is freely available as a downloadable file (http://ctdbase.org/downloads/#allanatomy). (B) CTD biocurators use an online tool (Interaction Entry Page) to generate interactions, which can be contextualized with CTD Anatomy terms. Anatomy curation is expandable, allowing terms from different branches to be multiplexed, adding specificity and enabling the data to be ultimately surveyed by users from multiple anatomical term pathways.

CTD Anatomy includes 1799 unique terms, of which 1326 (74%) are mapped to and enhanced with synonyms and cross-reference identifiers from 1227 Uberon and 265 CL terms (Fig. 1A). All merged synonyms and accession identifiers are searchable in CTD, and cross-references are provided on CTD Anatomy webpages to the respective ontologies using resource-specific accession identifiers. CTD Anatomy is freely available as a downloadable data file (http://ctdbase.org/downloads/#allanatomy).

### 3.2 Curating to CTD Anatomy

CTD Anatomy is used as a controlled vocabulary to annotate data in two curation modules: CTD Phenotype and CTD Exposure. CTD Phenotype describes chemical-induced, non-disease phenotypes from the scientific literature (Davis et al., 2013, Davis et al., 2018), typically derived from laboratory experiments, and these interactions are contextualized with anatomical terms to enable users to survey the system in which the toxicological event was reported. CTD Exposure provides real-world human exposure science statements (Grondin et al., 2016), using over 50 data fields to describe the exposure stressor (chemical, source category), human receptor demographics (description, age, gender, smoking status, race), exposure event (methods, biomarker assay, geographic location, statistics), and outcome (phenotype, disease). For biomarker measurements, CTD Exposure captures the concentrations of environmental chemicals assayed in human fluids, tissues, and cells, and for exposure outcomes, stressor-induced phenotypes are contextualized with anatomy terms (Grondin et al., 2018).

Multiple anatomy terms can be combined to provide greater specificity, such as linking together individual terms to annotate a chemical-induced phenotype reported in the mitochondria of hindlimb muscle fibroblasts (Fig. 1B). This multiplexing allows for exponential combinations, such as annotating cell types (e.g., Epithelial Cells) to any tissue (e.g., Lung, Kidney, Liver, Aorta) without the need to create individualized cell sub-types for every organ. Currently, over 880 anatomy terms are used to help contextualize more than 255,000 chemical-phenotype interactions and 167,000 exposure statements in CTD (http://ctdbase.org/about/dataStatus.go).

### 3.3 Accessing and viewing CTD Anatomy

**Fig. 2:**
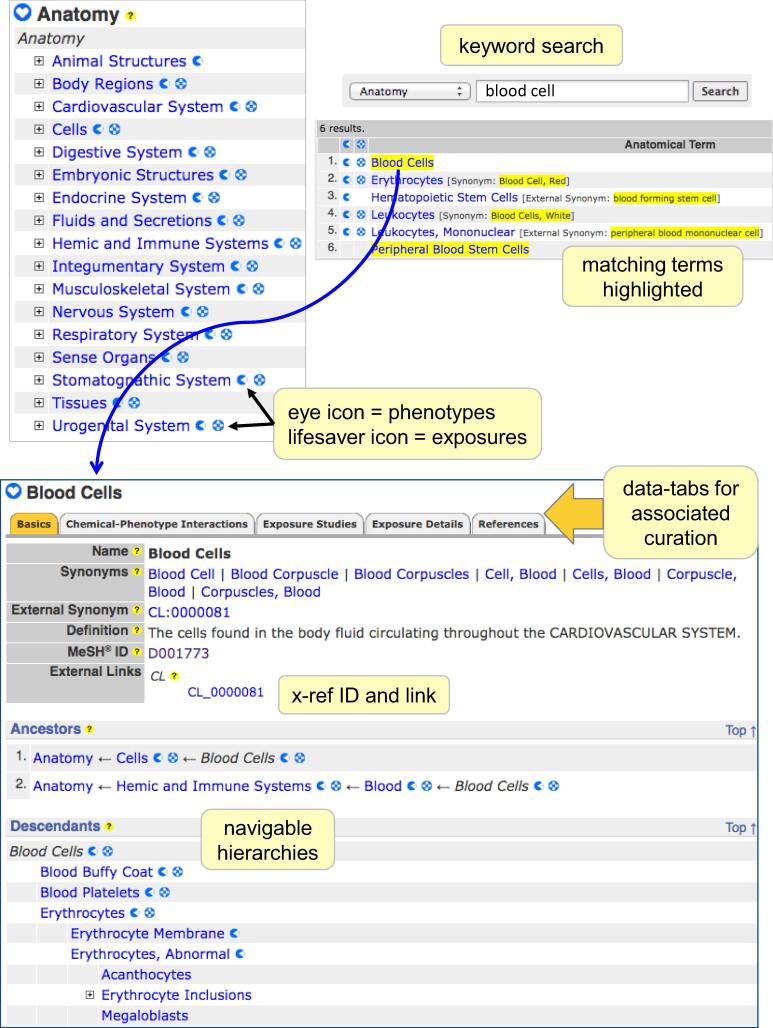
Accessing and viewing CTD Anatomy. The drill-down main hierarchy for CTD Anatomy (http://ctdbase.org/voc.go?type=anatomy) is organized into 17 top-level categories, including animal structures, cells, body regions, fluids, sense organs, tissues and 10 physiological systems. Icons indicate the type of curated data available for each term (‘eye’ for chemical-induced phenotypes and ‘lifesaver’ for exposures); clicking any icon will take the user to the specific data-tab for that term. Alternatively, users can perform a keyword search (picking ‘Anatomy’ from the drop-list), and results highlight the query terms. Every anatomy webpage has five data-tabs. The default tab (“Basics”) provides a definition, list of synonyms and link-out accession IDs, and navigable hierarchy with ancestor and descendant terms.

CTD Anatomy webpages enable users to survey and explore the associated curated data sets from an anatomical perspective and are now seamlessly integrated as a component of the database. Terms can be accessed either by drilling down the navigable main hierarchy (http://ctdbase.org/voc.go?type=anatomy) or by using the CTD Keyword Search Box (Fig. 2). The vocabulary is organized by 17 top-level categories, reflecting anatomical structures and regions, fluids and tissues, cells, sub-cellular components, and physiological systems. It is a navigable hierarchy, displaying both “Ancestors” (parent) and “Descendants” (children) terms. Nodes are indented to indicate their relative level in the displayed tree, and a node marked by a plus-symbol has descendants in at least one of the hierarchical paths displayed on the current page. Granular terms often map to multiple physiological systems because CTD Anatomy is structured as a polyhierarchic tree in which a term may appear as a node in more than one branch, and a term may have different descendant terms in each branch in which it appears. For example, searching with the phrase “blood cells” brings back the page Blood Cells (Fig. 2), which traces back to two ancestor terms (Cells and Hemic and Immune Systems) and down to multiple descendants (e.g., Blood Buffy Coat, Blood Platelets, Erythrocytes, etc.) and is externally linked (CL_0000081) to the corresponding CL term “blood cell”.

CTD Anatomy webpages are partitioned into five data-tabs that parallel the content seen on other CTD pages: “Basics” lists the official term name, definition, synonyms, accession identifier, and external links; “Chemical-Phenotype Interactions” provides a tabular format of the curated chemical-induced phenotypes interaction that can be sorted by chemical, phenotype, co-mentioned term, organism, anatomy, and gene inference network column headers, as previously described (Davis et al., 2020); “Exposure Studies” provides a summary statement and a broad view of the reported exposure project, while “Exposure Details” itemizes marker levels, measurements, statistics, and outcome relationships (Grondin et al., 2016); and “References” collates all curated articles and their cited terms.

### 3.4 Using CTD Anatomy

In the examples below, we demonstrate how CTD Anatomy pages can be used to survey and leverage curated chemical-phenotype and exposure data sets, and how this information can help apprise and inform environmental health studies and initiate the generation of testable hypotheses.

#### 3.4.1 Example 1: surveying chemical toxicities via anatomy

**Fig. 3:**
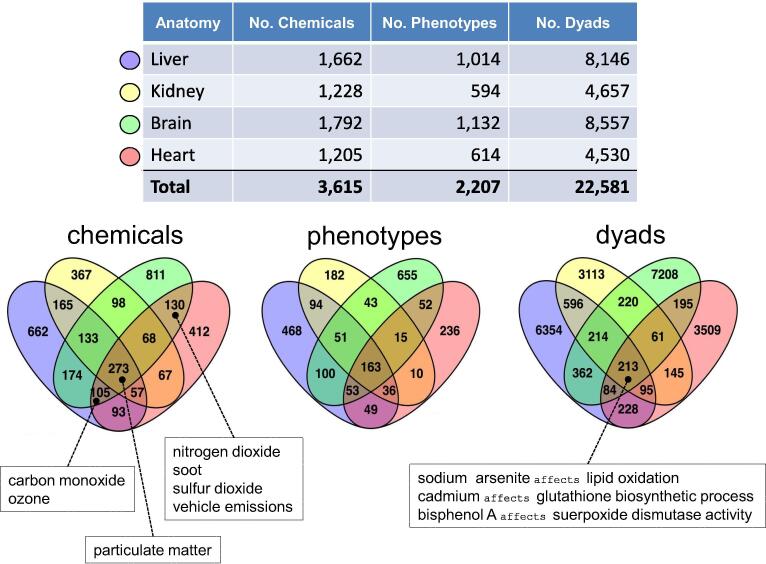
Using CTD Anatomy to identify organ toxicities. The numbers and distribution of unique chemicals, phenotypes, and chemical-phenotype dyads are shown for liver, kidney, brain, and heart. Venn analysis discovers unique and shared data types for each anatomical location, such as ambient air pollutants (carbon monoxide, ozone, nitrogen dioxide, soot, sulfur dioxide, vehicle emissions, and particulate matter) affecting different subsets, and environmental chemicals (sodium arsenite, cadmium, bisphenol A) affecting toxicities related to basic cellular metabolism in all four organs.

Improving the prediction of chemical toxicity is a common goal for both environmental health research and pharmaceutical drug development (Davis et al., 2013, Pelletier et al., 2016). Exploring CTD chemical-phenotype interactions from an anatomical perspective facilitates the identification of system-specific chemical actions and phenotypes, which, in turn, can help inform toxicity, side effects, and potential areas of interaction between environmental chemicals and therapeutic drugs (Fardel et al., 2012). CTD Anatomy permits easy visualization of the distribution of chemical-induced phenotypes (i.e., toxicities) for liver, kidney, brain, and heart (Fig. 3). At CTD, a chemical-phenotype interaction is constructed as a dyad, consisting of a chemical and the phenotype that it modulates. We compared not just the number and type of unique chemicals and phenotypes independently, but also the chemical-phenotype dyads.

In total, 3615 chemicals, 2207 phenotypes, and 22,581 dyads are distributed across the four anatomical terms, with many environmental chemicals, such as air pollutants (carbon monoxide, nitrogen dioxide, particular matter, ozone, soot, vehicle emissions, and sulfur dioxide) affecting all four systems (Fig. 3). As well, each organ has unique data not present in any of the other three, providing potential sets of system-specific chemical actions and modulated processes. Some of the toxicities (and the chemicals that induce them) unique to the liver include effects on lipid homeostasis (quercetin, tobacco smoke, diisononyl phthalate) and hepatic stellate cell activation (lipopolysaccharides, tetramethylpyrazine, curcumin, methyl ferulate); kidney-restricted phenotypes include aberrations in glomerular basement membrane development (dietary fats, caffeine), ferroptosis (vitamin E), misregulation of glomerular filtration (particular matter, cadmium, lead, nitric oxide), and renal tubular secretion (rifampin, notoginsenoside R1); brain-restricted toxicities include defects in dopamine metabolism (rotenone, geraniol, baicalein), excitatory postsynaptic potential (nicotine, sulfur dioxide, lead acetate), and neurogenesis (cadmium, manganese, perfluorooctane sulfonic acid); and heart toxicities include modulations in cardiac muscle cell contraction (bisphenol A, caffeine, paraquat), heart rate (endotoxins, fentanyl, methylatropine), and heart looping (flame retardants, mannitol, tetrachlorodibenzodioxin). Many chemical-phenotype dyads are associated with all four tissues, and these represent toxicities in basic metabolic processes common to most cell-types, such as lipid oxidation, glutathione metabolism, and superoxide dismutase activity.

Surveying chemicals and outcomes from an anatomical perspective provides insight about potential chemical-induced toxicities that interact in different, shared, and unique physiological systems (Taboureau et al., 2020). For example, the ambient air pollutant particulate matter affects over 30 phenotypes in the heart. Interestingly, many of these induced toxicities (e.g., calcium-mediated signaling, activation of protein kinase B, cardiac muscle contraction, regulation of heart rate) are modulated in this same tissue by known cardiovascular therapeutics (e.g., propranolol, esmolol, nifedipine, nitroprusside, and prazosin). Molecular mechanistic models now can be developed to study potential interactions between air pollutants and pharmaceutical drugs to explore how these interactions could affect human health in medicated patients (Tumiatti et al. 2018). A critical facet, and challenge, to understanding environmental health is to recognize the influence of toxicant mixtures and combined exposures (Martins et al., 2019). Towards that end, identifying system-specific chemical-induced phenotypes (and the genes shared between the dyads) should help facilitate the development of co-exposure mechanistic hypotheses for potential interactions between two or more chemicals that synergize in the same tissue at low doses to produce an outcome not seen in a single exposure, as described for interactions between bisphenol A and environmental chemicals (Sonavane and Gassman, 2019).

**Fig. 4:**
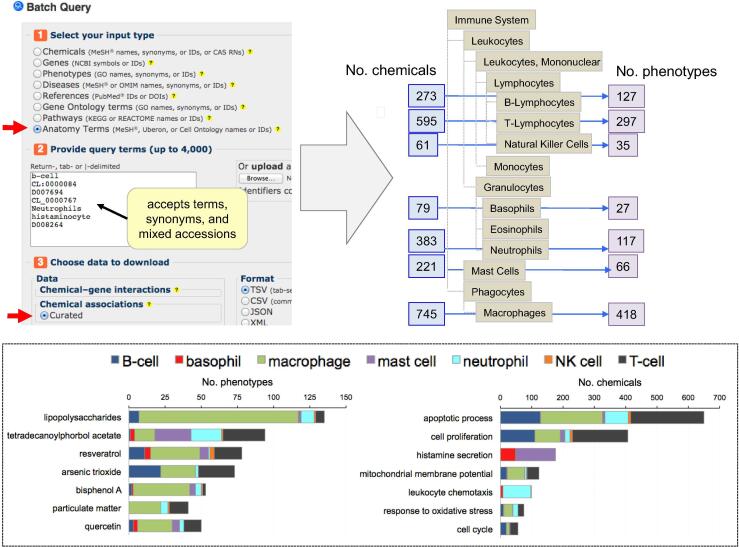
Using CTD Anatomy to identify cell toxicities. CTD’s Batch Query (http://ctdbase.org/tools/batchQuery.go) accepts anatomy terms as input values (official terms, synonyms, or interoperable accessions). Downloading the associated chemical-phenotype interaction data identifies the unique number of chemicals and induced phenotypes associated with each cell type to enable users, for example, to build immunotoxicology models at the cell level. A schematic drawing of the immune system hierarchy is overlaid with the retrieved number of unique chemicals and phenotypes for each surveyed immune cell. Some of the top chemical toxicants (e.g. lipopolysaccharides, resveratrol, arsenic trioxide, bisphenol A) and top induced-phenotypes (e.g., apoptosis, cell proliferation, histamine secretion, cell cycle) are graphed based upon the type of immune cell in which the toxicity was reported (bottom box).

In addition to tissues and organs, cells can be surveyed for chemical-induced toxicities. For example, multiple terms can be searched simultaneously using CTD’s Batch Query (http://ctdbase.org/tools/batchQuery.go) to identify chemical-phenotype dyads associated with a variety of specific immune cells (Fig. 4). Here, results show a strong chemical influence of lipopolysaccharides on macrophages (triggering over 100 phenotypes) and tetradecanoylphorbol acetate on mast cells (25 phenotypes). The most reported chemical-induced toxicities relate to apoptosis and cell proliferation (in all immune cells) and abnormal histamine secretion (in basophils and mast cells), mitochondrial membrane potential, and cell cycle irregularities (Fig. 4). This strategy elucidates chemical toxicities in different immune system cell compartments and might help build models for environmental toxicology (Thompson et al., 2015).

#### 3.4.2 Example 2: informing environmental diseases with anatomical-based phenotypes

Anatomy curation enables users to discover environmental factors and molecular mechanisms associated with environmental diseases without a priori knowledge of any chemical, gene, phenotype, or disease outcome. Two cases are described showing how CTD Anatomy can be used to inform the understanding of the pathogenesis of environmental diseases.

**Fig. 5:**
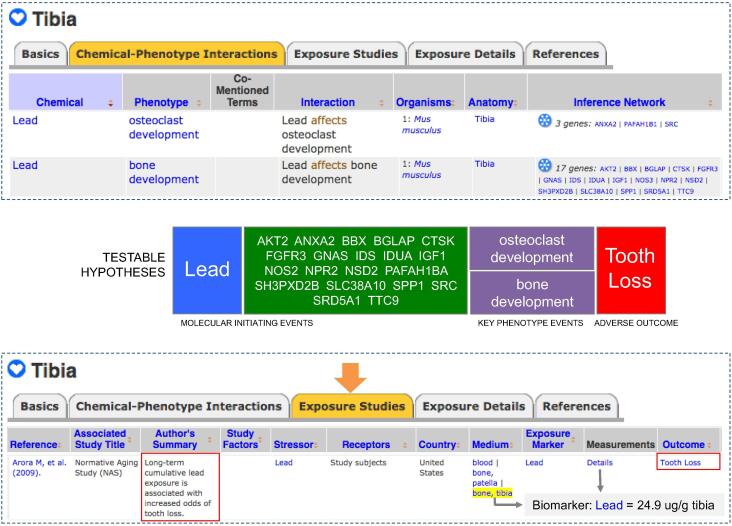
Using CTD Anatomy to help inform exposure data. The same anatomy vocabulary is used to annotate both chemical-phenotype and exposure data, and CTD Anatomy webpages coalesce this heterogeneous information, allowing experimental results to be connected to and help inform real-world outcomes. On the Tibia webpage, the “Chemical-Phenotype Interactions” data-tab (top) describes the role of lead in osteoclast and bone development in the tibia of mice, while the “Exposure Studies” data-tab (bottom) report how long-term cumulative lead exposure in humans (24.9 µg per gram tibia) correlates with excessive tooth loss. Users can leverage these data to generate testable hypotheses (middle) for potential molecular mechanisms and adverse outcome pathways to help fill in the knowledge gaps for environmental health; in this example, lead interacts with 20 known genes (from the Inference Network) to modulate bone phenotypes, which in turn could be related to tooth loss.

First, CTD Phenotype and CTD Exposure are two independent curation modules that use CTD Anatomy for annotations, connecting experimental laboratory results with real-world exposure information that, in turn, can help inform environmental health studies. For example, a shared anatomy annotation (Tibia) connects two independent findings: a mouse study showing how lead modulates osteoclast and bone development phenotypes in the tibia (Beier et al., 2016), and a human exposure study (Arora et al., 2009) correlating increased lead levels in the tibia with excessive tooth loss (Fig. 5). The shared entities (Lead and Tibia) link these two independent studies, allowing anatomical-based phenotypes to help inform a hypothetical environmental health study; here, testable hypotheses about potential molecular mechanisms can be generated for lead interacting with any of the 20 genes/proteins to influence bone phenotypes (osteoclast and bone development) contributing to tooth loss in exposed humans; such data models can be used to help propose and refine formal adverse outcome pathways (Ankley and Edwards, 2018).

**Fig. 6:**
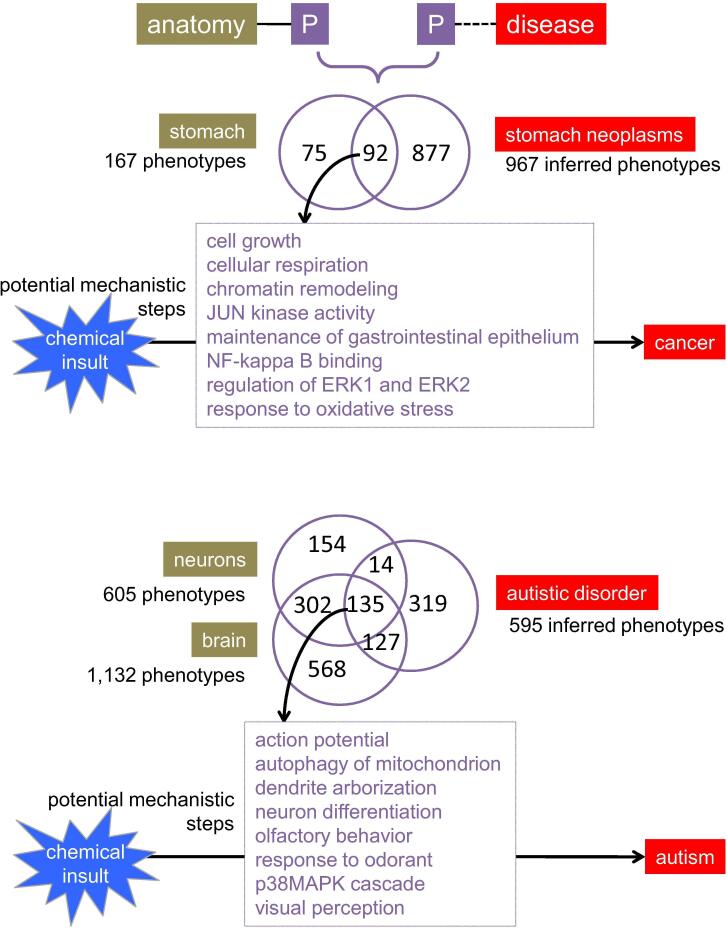
Using CTD Anatomy to prioritize phenotypes for environmental diseases. Phenotypes annotated with anatomy terms can be used to help inform anatomical-based environmental diseases. Here, CTD Anatomy describes 167 chemical-induced phenotypes studied in stomach tissue/cells. Comparing these 167 anatomical phenotypes with the 967 phenotypes inferred to stomach cancer identifies a subset of 92 that can be prioritized for generating mechanistic hypotheses (boxes), since they represent phenotypes that are both known to be chemical-induced in stomach tissue as well as being inferred to stomach cancer (by shared chemicals and genes). Similar analysis can be performed for other environmental diseases, like autism, filtered against phenotypes for disease-relevant anatomical terms (e.g., neurons and brain).

Second, anatomical-based phenotypes can help inform and prioritize mechanistic models for anatomical-based diseases, such as tissue-specific cancers or brain-related disorders (Fig. 6). For example, 967 phenotypes can be inferred to Stomach Neoplasms based upon both the shared set of chemicals (Chemical Inference Network, CIN) and genes (Gene Inference Network, GIN). One method to help prioritize this set is to filter them by phenotypes independently reported to be chemically induced in the corresponding tissue, i.e., stomach. CTD’s anatomy page for Stomach provides 167 chemical-induced phenotypes reported in stomach cells/tissue, and when compared against the 967 inferred phenotypes, refines the list to 92 phenotypes that are both inferred to stomach cancer by CIN and GIN and shown to occur in stomach cells/tissue. These prioritized phenotypes provide potential mechanistic steps to build testable models for chemical-induced stomach cancer, such as response to oxidative stress, altered cellular signaling (JUN kinase, NF-kappaB, and ERK1/ER2), and de-stabilization of cell growth and gastrointestinal epithelium (Fig. 6). Many of these computed phenotypes are validated in the literature as playing a role in chemical-induced stomach cancer, including altered cell signaling by NF-kappaB (Sokolova and Naumann, 2017), ERK1/2 (Khoi et al., 2014), and JUN (Li et al., 2013). This is not to suggest that the other phenotypes do not necessarily play a role in stomach cancer. The prioritized phenotypes enable users to initiate the design of testable hypotheses, especially since these 92 refined phenotypes also have associated CIN and GIN. This permits the construction of chemical-gene-phenotype-disease (CGPD)-tetramers that fill in knowledge gaps and help assemble chemical-disease pathways (Davis et al., 2020). Similarly, the inferred phenotypes for non-cancer environmental diseases like autistic disorder can be first filtered with phenotypes from tissues predicted to play a role in the disease (e.g., neurons and brain) to refine potential mechanistic processes (e.g., action potentials, autophagy, dendrite arborization, olfactory behavior, etc.) to design environmental models for autism (Fig. 6).

#### 3.4.3 Example 3: tissue-based exposomes

**Fig. 7:**
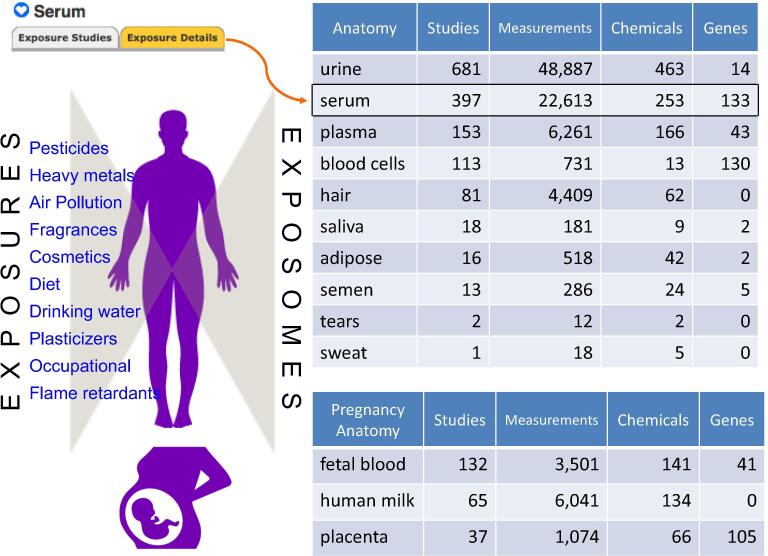
Using CTD Anatomy to survey exposomes. The “Exposure Studies” and “Exposure Details” data-tabs on CTD Anatomy pages coalesce (from different publications) all curated measurements for chemicals and gene/protein markers assayed in humans in response to exposures, such as pesticides, air pollution, environmental contaminants, household products, diet, flame retardants, etc. In human serum, 397 exposure studies have been curated that report more than 22,000 measured values for 250 chemicals and 130 genes/proteins. Anatomy terms related to pregnancy, such as human milk, provide a snapshot of the early life exposome.

The ‘exposome’ concept represents all exposures of an individual (e.g., chemical, biological, physical, behavioral, societal, psychological, etc.) across their life course since conception and how those exposures relate to health effects and outcomes (Wild, 2005). One facet to understanding exposomes and their relationship to human health is to identify and quantify the metabolites, biomarkers, and biological processes in response to environmental chemical exposures from air pollution, diet, cosmetics, fragrances, drinking water, flame retardants, etc. (Vermeulen et al., 2020). Measuring and defining anatomy-specific exposomes, such as for blood (Rappaport et al., 2014), urine (Gao, 2013;), lung (Wheelock and Rappaport, 2020), and kidney (Dupre et al., 2020), has been proposed to help understand the gene-environment interactions associated with human environmental diseases. In the CTD Exposure module, chemical and genes/protein biomarkers are curated for exposure events from the literature (Grondin et al., 2016), and these measurements are annotated with anatomical terms for the biological media assayed. These media, in turn, are integrated to CTD Anatomy, allowing exposure measurements captured from different articles to be coalesced from an anatomical perspective (Fig. 7). Thus, users can explore on-the-fly tissue-specific exposomes for any part of the human body, including well-studied fluids such as urine (681 studies, 48,800 measurements, 463 chemicals) and serum (397 studies, 22,600 measurements, 253 chemicals), as well as less commonly studied exposomes, e.g., semen (24 chemicals), saliva (9 chemicals), and sweat (5 chemicals).

Anatomical terms related to pregnancy (e.g., human milk, fetal blood, placenta) allow the infant/newborn exposome to be surveyed for early life events. For example, more than 15 polybrominated flame retardants have been detected in breast milk (http://ctdbase.org/detail.go?type=anatomy&acc=D008895&view=expConsol). Median values for the retardant PBDE-47 range between 0.1 and 32 ng per gram lipid, as reported by 26 studies from 13 countries, with one study correlating PBDE-47 levels in breast milk with congenital cryptorchidism in newborn boys (Main et al., 2007). As well, numerous polychlorinated biphenyls (PCBs) have been measured in the fetal blood exposome (http://ctdbase.org/detail.go?type=anatomy&acc=D005312&view=expConsol), with several correlating to decreased developmental growth (PCB-138), fetal growth retardation (PCB-153), and decreased cognition (PCB-118). These exposome chemicals can be further explored in CTD to discover interacting genes and induced phenotypes to help construct testable models for prenatal exposure delayed effects.

### 3.5 Bringing other resources into the chemical environment via shared anatomy

Gene-environment interactions are paramount to understanding biological traits and diseases, and while numerous public databases focus on genetic and genomic information, relatively fewer resources catalog the environmental components or their influences (Mattingly, 2009). Using informatics to integrate environmental exposure data with human genomic resources is a community-recognized need and challenge for elucidating the environmental risk factors for disease (Thessen et al., 2020). CTD currently uses community-accepted vocabularies and identifiers when curating chemical, gene, phenotype, disease, and taxon data, and these can be used as accession points for external resources (Davis et al., 2019). Adding interoperable identifiers for Uberon and CL into CTD Anatomy provides yet another way to establish connections between CTD and external resources, further enabling CTD chemicals and environmental exposures to be brought into genomic databases to explore gene-environment interactions. For example, the CTD Anatomy page for Lung is identified by the primary accession MeSH:D008168 (http://ctdbase.org/detail.go?type=anatomy&acc=D008168) but includes the matched external identifier UBERON:0002048 to promote interoperability with resources that employ other anatomical vocabularies. UBERON:0002048 is used to annotate ‘lung’ to a plethora of genes, transcriptomes, and genomic regions in resources such as Monarch Initiative (Shefchek et al., 2020), FANTOM5 (Kawaji et al., 2017), ENCODE (Malladi et al., 2015), GeneWeaver (Baker et al., 2016), and GEO DataSets (Barrett et al., 2013), among others. CTD’s content for Lung includes 1300 chemicals affecting 800 phenotypes curated from more than 1600 articles, and this information can be integrated with these genomic databases via the shared interoperable accession identifier for anatomy. Discovering and combining diverse public data sources helps fill in knowledge gaps and facilitates the computational design of pathways to support systems toxicology applications (Kosnik et al., 2019, Ives et al., 2017) and environmental exposure models (Thessen et al., 2020). CTD’s chemical-induced toxicities, already identified by specific anatomical sites, should help further support and refine these models and molecular mechanisms.

## 4 Conclusion

We present and describe CTD Anatomy, a new type of contextualizing information that allows users to explore curated chemical-phenotype and exposure data from a unique anatomical perspective. We provide numerous examples highlighting how this feature can be used to approach environmental health studies, including identifying tissue-specific toxicities and immune cell dysregulation, generating testable hypotheses that fill in knowledge gaps to connect lead exposure to excessive tooth loss, prioritizing tissue-related phenotypes for environmental diseases such as stomach cancer and autism, surveying tissue-specific and pregnancy-related exposomes, and exploiting the interoperability of anatomy terms to exchange data with and add content to external genetic/genomic resources to help integrate them into the chemical environment provided by CTD.

Currently, users can leverage CTD anatomy data to survey chemical-induced toxicities, either by anatomy-specific webpages (http://ctdbase.org/voc.go?type=anatomy), batch queries (http://ctdbase.org/tools/batchQuery.go), or single queries (http://ctdbase.org/query.go?type=phenotype). Additional new tools are planned, including a web-based application that generates on-the-fly CGPD-tetramers (Davis et al., 2020), enabling a user to input a term-of-interest (i.e., chemical, gene, phenotype, or disease) and the tool automatically computes the possible CGPD-tetramers to fill in the knowledge gaps. Selecting an anatomy term as an additional parameter will refine the output to tissue-specific pathways.

## Funding

This work was supported by the 10.13039/100000066National Institute of Environmental Health Sciences (NIEHS) grant numbers ES014065, ES023788, ES019604, and ES025128. The content is solely the responsibility of the authors and does not necessarily represent the official views of the 10.13039/100000002National Institutes of Health.

## CRediT authorship contribution statement

**Allan Peter Davis:** Conceptualization, Methodology, Formal analysis, Data curation, Writing - original draft, Writing - review & editing. **Thomas C. Wiegers:** Software, Methodology, Formal analysis, Writing - review & editing, Writing - review & editing. **Jolene Wiegers:** Software, Methodology, Formal analysis, Writing - review & editing. **Cynthia J. Grondin:** Data curation, Writing - review & editing. **Robin J. Johnson:** Data curation, Writing - review & editing. **Daniela Sciaky:** Data curation, Writing - review & editing. **Carolyn J. Mattingly:** Project administration, Funding acquisition, Writing - review & editing.

## Declaration of Competing Interest

The authors declare that they have no known competing financial interests or personal relationships that could have appeared to influence the work reported in this paper.

## References

[b0005] Amberger J.S., Bocchini C.A., Scott A.F., Hamosh A. (2019). OMIM.org: leveraging knowledge across phenotype-gene relationships. Nucleic Acids Res..

[b0010] Ankley G.T., Edwards S.W. (2018). The adverse outcome pathway: a multifaceted framework supporting 21st century toxicology. Curr. Opin. Toxicol..

[b0015] Arora M., Weuve J., Weisskopf M.G., Sparrow D., Nie H., Garcia R.I., Hu H. (2009). Cumulative lead exposure and tooth loss in men: the normative aging study. Environ. Health Perspect..

[b0020] Ashburner M., Ball C.A., Blake J.A., Botstein D., Butler H., Cherry J.M., Davis A.P., Dolinski K., Dwight S.S., Eppig J.T. (2000). Gene Ontology: tool for the unification of biology. Nat. Genet..

[b0025] Baker E., Bubier J.A., Reynolds T., Langston M.A., Chesler E.J. (2016). GeneWeaver: data driven alignment of cross-species genomics in biology and disease. Nucleic Acids Res..

[b0030] Barabasi A.-L., Menichetti G., Loscalzo J. (2020). The unmapped chemical complexity of our diet. Nat. Food..

[b0035] Barrett T., Wilhite S.E., Ledoux P., Evangelista C., Kim I.F., Tomashevshy M., Marshall K.A., Phillippy K.H., Sherman P.M., Holko M. (2013). NCBI GEO: archive for functional genomics data sets–update. Nucleic Acids Res..

[b0040] Beier E.E., Holz J.D., Sheu T.-J., Puzas J.E. (2016). Elevated lifetime lead exposure impedes osteoclast activity and produces an increase in bone mass in adolescent mice. Toxicol. Sci..

[b0045] Brennan G.P., Bauer S., Engel T., Jimenez-Mateos E.M., Del Gallo F., Hill T.D.M., Connolly N.M.C., Costard L.S., Neubert V., Salvetti B. (2020). Genome-wide microRNA profiling of plasma from three different animal models identifies biomarkers of temporal lobe epilepsy. Neurobiol. Dis..

[b0050] Cheng S., Wen Y., Ma M., Zhang L., Liu L., Qi X., Cheng B., Liang C., Li P., Kafle O.P., Zhang F. (2020). Identifying 5 common psychiatric disorders associated chemicals through integrative analysis of genome-wide association study and chemical-gene interaction datasets. Schizophr. Bull..

[b0055] Coletti M.H., Bleich H.L. (2001). Medical subject headings used to search the biomedical literature. J. Am. Med. Inform. Assoc..

[b0060] Dahdul W., Dececchi T.A., Ibrahim N., Lapp H., Mabee P. (2015). Moving the mountain: analysis of the effort required to transform comparative anatomy into computable anatomy. Database (Oxford).

[b0065] Davis A.P., Murphy C.G., Rosenstein M.C., Wiegers T.C., Mattingly C.J. (2008). The Comparative Toxicogenomics Database facilitates identification and understanding of chemical-gene-disease associations: arsenic as a case study. BMC Med. Genomics.

[b0070] Davis A.P., Wiegers T.C., Rosenstein M.C., Murphy C.G., Mattingly C.J. (2011). The curation paradigm and application tool used for manual curation of the scientific literature at the Comparative Toxicogenomics Database. Database (Oxford).

[b0075] Davis A.P., Wiegers T.C., Rosenstein M.C., Mattingly C.J. (2012). MEDIC: a practical disease vocabulary used at the Comparative Toxicogenomics Database. Database (Oxford).

[b0080] Davis A.P., Wiegers T.C., Roberts P.M., King B.L., Lay J.M., Lennon-Hopkins K., Sciaky D., Johnson R., Keating H., Greene N. (2013). A CTD-Pfizer collaboration: manual curation of 88,000 scientific articles text mined for drug-disease and drug-phenotype interactions. Database (Oxford).

[b0085] Davis A.P., Grondin C.J., Lennon-Hopkins K., Saraceni-Richards C., Sciaky D., King B.L., Wiegers T.C., Mattingly C.J. (2015). The Comparative Toxicogenomics Database's 10th year anniversary: update 2015. Nucleic Acids Res..

[b0090] Davis A.P., Wiegers T.C., Wiegers J., Johnson R.J., Sciaky D., Grondin C.J., Mattingly C.J. (2018). Chemical-induced phenotypes at CTD help inform the predisease state and construct adverse outcome pathways. Toxicol. Sci..

[b0095] Davis A.P., Wiegers J., Wiegers T.C., Mattingly C.J. (2019). Public data sources to support systems toxicology applications. Curr. Opin. Toxicol..

[b0100] Davis A.P., Wiegers T.C., Grondin C.J., Johnson R.J., Sciaky D., Wiegers J., Mattingly C.J. (2020). Leveraging the Comparative Toxicogenomics Database to fill in knowledge gaps for environmental health: a test case for air pollution-induced cardiovascular disease. Toxicol. Sci..

[b0105] Davis A.P., Grondin C.J., Johnson R.J., Sciaky D., Wiegers J., Wiegers T.C., Mattingly C.J. (2021). Comparative Toxicogenomics Database (CTD): update 2021. Nucleic Acids Res..

[b0110] Diehl A.D., Meehan T.F., Bradford Y.M., Brush M.H., Dahdul W.M., Dougall D.S., He Y., Osumi-Sutherland D., Ruttenberg A., Sarntivijai S. (2016). The Cell Ontology 2016: enhanced content, modularization, and ontology interoperability. J. Biomed. Semantics.

[b0115] Dupre T.V., Schnellmann R.G., Miller G.W. (2020). Using the exposome to address gene-environment interactions in kidney disease. Nat. Rev. Nephrol..

[b0120] Fardel O., Kolasa E., Vee M.L. (2012). Environmental chemicals as substrates, inhibitors or inducers of drug transporters: implication for toxicokinetics, toxicity and pharmacokinetics. Expert Opin. Drug Metab. Toxicol..

[b0125] Gao Y. (2013). Urine – an untapped goldmine for biomarker discovery?. Sci. China Life Sci..

[b0130] Gokhman D., Kelman G., Amartely A., Gershon G., Tsur S., Carmel L. (2017). Gene ORGANizer: linking genes to the organs they affect. Nucleic Acids Res..

[b0135] Gong L., Luo Z., Tang H., Tan X., Xie L., Lei Y., He C., Ma J., Han S. (2020). Integrative, genome-wide association study identifies chemicals associated with common women's malignancies. Genomics.

[b0140] Grondin C.J., Davis A.P., Wiegers T.C., King B.L., Wiegers J.A., Reif D.M., Hoppin J.A., Mattingly C.J. (2016). Advancing exposure science through chemical data curation and integration in the Comparative Toxicogenomics Database. Environ. Health Perspect..

[b0145] Grondin C.J., Davis A.P., Wiegers T.C., Wiegers J.A., Mattingly C.J. (2018). Accessing an expanded exposure science module at the Comparative Toxicogenomics Database. Environ. Health Perspect..

[b0150] Ives C., Campia I., Wang R.-L., Wittwehr C., Edwards S. (2017). Creating a structured AOP knowledgebase via ontology-based annotations. Appl. In Vitro Toxicol..

[b0155] Kafle O.P., Cheng S., Ma M., Li P., Cheng B., Zhang L., Wen Y., Liang C., Qi X., Zhang F. (2020). Identifying insomnia-related chemicals through integrative analysis of genome-wide association studies and chemical-genes interaction information. Sleep.

[b0160] Kawaji H., Kasukawa T., Forrest A., Carninci P., Hayashizaki Y. (2017). The FANTOM5 collection, a data series underpinning mammalian transcriptome atlases in diverse cell types. Sci. Data.

[b0165] Kitsak M., Sharma A., Menche J., Guney E., Ghiassian S.D., Loscalzo J., Barabasi A.-L. (2016). Tissue specificity of human disease module. Sci. Rep..

[b0170] Khoi P.N., Xia Y., Lian S., Kim H.D., Kim D.H., Joo Y.E., Chay K.-O., Kim K.K., Jung Y.D. (2014). Cadmium induces urokinase-type plasminogen activator receptor expression and the cell invasiveness of human gastric cancer cells via the ERK-1/2, NF-kB, and AP-1 signaling pathways. Int. J. Oncol..

[b0175] Kosnik M.B., Planchart A., Marvel S.W., Reif D.M., Mattingly C.J. (2019). Integration of curated and high-throughput screening data to elucidate environmental influences on disease pathways. Comput. Toxicol..

[b0180] Li R., Tian J., Li W., Xie J. (2013). Effects of 2-amino-1-methyl-6-phenylimidazo [4, 5-b] pyridine (PhIP) on histopathology, oxidative stress, and expression of c-fos, c-jun and p16 in rat stomachs. Food Chem. Toxicol..

[b0185] Lu X., Fraszczyk E., van der Meer T.P., van Faassen M., Bloks V.W., Kema I.P., van Beek A.P., Li S., Franke L., Westra H.-J. (2020). An epigenome-wide association study identifies multiple DNA methylation markers of exposure to endocrine disruptors. Environ. Int..

[b0190] Main K.M., Kiviranta H., Virtanen H.E., Sundqvist E., Tuomisto J.T., Tuomisto J., Vartiainen T., Skakkebaek N.E., Toppari J. (2007). Flame retardants in placenta and breast milk and cryptorchidism in newborn boys. Environ. Health Perspect..

[b0195] Malladi V.S., Erickson D.T., Podduturi N.R., Rowe L.D., Chan E.T., Davidson J.M., Hitz B.C., Ho M., Lee B.T., Miyasato S. (2015). Ontology application and use at the ENCODE DCC. Database (Oxford).

[b0200] Martins C., Dreij K., Costa P.M. (2019). The state-of-the art of environmental toxicogenomics: challenges and perspectives of “omics” approaches directed to toxicant mixtures. Int. J. Environ. Res. Public Health.

[b0205] Mattingly C.J. (2009). Chemical databases for environmental health and clinical research. Toxicol. Lett..

[b0210] Mungall C.J., Torniai C., Gkoutos G.V., Lewis S.E., Haendel M.A. (2012). Uberon, an integrative multi-species anatomy ontology. Genome Biol..

[b0215] Pelletier D., Wiegers T.C., Enayetallah A., Kibbey C., Gosnik M., Koza-Taylor P., Mattingly C.J., Lawton M. (2016). ToxEvaluator: an integrated computational platform to aid the interpretation of toxicology study-related findings. Database (Oxford).

[b0220] Rappaport S.M. (2016). Genetic factors are not the major causes of chronic diseases. PLoS ONE.

[b0225] Rappaport S.M., Barupal D.K., Wishart D., Vineis P., Scalbert A. (2014). The blood exposome and its role in discovering causes of disease. Environ. Health Perspect..

[b0230] Sayers E.W., Beck J., Brister J.R., Bolton E.E., Canese K., Comeau D.C., Funk K., Ketter A., Kim S., Kimchi A. (2020). Database resources of the National Center for Biotechnology Information. Nucleic Acids Res..

[b0235] Shefchek K.A., Harris N.L., Gargano M., Matentzoglu N., Unni D., Brush M., Keith D., Conlin T., Vasilevsky N., Zhang X.A. (2020). The Monarch Initiative in 2019: an integrative data and analytic platform connecting phenotypes to genotypes across species. Nucleic Acids Res..

[b0240] Smith C.J., Perfetti T.A. (2020). Exposure to chemicals formed from natural processes is ubiquitous. Toxicol. Res. Appl..

[b0245] Sokolova O., Naumann M. (2017). NF-kB signaling in gastric cancer. Toxins (Basel)..

[b0250] Sonavane M., Gassman N.R. (2019). Bisphenol A co-exposure effects: a key factor in understanding BPA’s complex mechanism and health outcomes. Crit. Rev. Toxicol..

[b0255] Taboureau O., El M’Selmi W., Audouze K. (2020). Integrative systems toxicology to predict human biological systems affected by exposure to environmental chemicals. Toxicol. Appl. Pharmacol..

[b0260] Tan X., Tang H., Gong L., Xie L., Lei Y., Luo Z., He C., Ma J., Han S. (2020). Integrating genome-wide association studies and gene expression profiles with chemical-genes interaction networks to identify chemicals associated with colorectal cancer. Front. Genet..

[b0265] Thessen A.E., Grondin C.J., Kulkarni D., Brander S., Truong L., Vasilevsky N.A., Callahan T.J., Chan L.E., Westra B., Willis M. (2020). Community approaches for integrating environmental exposures into human models of disease. Environ. Health Perspect..

[b0270] Thompson P.A., Khatami M., Baglole C.J., Sun J., Harris S.A., Moon E.-Y., Al-Mulla F., Al-Temaimi R., Brown D.G., Colacci A. (2015). Environmental immune disruptors, inflammation and cancer risk. Carcinogenesis.

[b0275] Tumiatti V., Fimognari C., Milelli A., Manucra D., Capello F., Gaddi A. (2018). Pollutants and Drugs: Interactions and Human Health. Clinical Handbook of Air Pollution-Related Diseases.

[b0280] Vermeulen R., Schymanski E.L., Barabasi A.-L., Miller G.W. (2020). The exposome and health: where chemistry meets biology. Science.

[b0285] Wang M., Zhao J., Wang Y., Mao Y., Zhao X., Huang P., Liu Q., Ma Y., Yao Y., Yang Z. (2020). Genome-wide DNA methylation analysis reveals significant impact of long-term ambient air pollution exposure on biological functions related to mitochondria and immune response. Environ. Pollut..

[b0290] Wheelock C.E., Rappaport S.M. (2020). The role of gene-environment interactions in lung disease: the urgent need for the exposome. Eur. Respir. J..

[b0295] Wild P.W. (2005). Complementing the genome with an “exposome”: the outstanding challenge of environmental exposure measurement in molecular epidemiology. Cancer Epidemiol. Biomarkers Prev..

[b0300] Wilkinson M.D., Dumontier M., Aalbersberg I.J.J., Appleton G., Axton M., Baak A., Blomberg N., Boiten J.-W., da Silva Santos L.B., Bourne P.E. (2016). The FAIR Guiding Principles for scientific data management and stewardship. Sci. Data.

